# Long Term Outcomes and Predictors of Reverse Remodelling After Cardiac Resynchronization Therapy Upgrade

**DOI:** 10.3390/medicina62030513

**Published:** 2026-03-10

**Authors:** Jakub Simka, Eva Cermakova, Rudolf Praus, Jiri Dokoupil, Jakub Stritecky, Ludek Haman, Filip Varhanik, Radek Pudil, Petr Parizek

**Affiliations:** 1 First Department of Internal Medicine, Cardiology and Angiology, University Hospital Hradec Kralove, 500 05 Hradec Kralove, Czech Republic; 2 Faculty of Medicine in Hradec Kralove, Charles University, 500 03 Hradec Kralove, Czech Republic

**Keywords:** cardiac resynchronization therapy, heart failure, pacing induced cardiomyopathy, upgrade

## Abstract

*Background and Objectives*: Upgrades to cardiac resynchronization therapy (CRT) account for approximately one quarter of all CRT indications. Although recent clinical trials have demonstrated significant clinical benefits of upgrade procedures, data on the long-term clinical effects of CRT upgrades remain limited. This study aimed to evaluate long-term clinical, echocardiographic, and device-related outcomes after CRT upgrade and to determine predictors of left ventricular reverse remodelling. *Materials and Methods*: A total of 97 patients underwent CRT upgrade at a tertiary referral centre, including 57 patients upgraded to CRT with pacemaker (CRT-P) and 40 to CRT with defibrillator (CRT-D). *Results*: During a 5-year follow-up period, 46 patients (47%) died from any cause. Appropriate device therapy was recorded in 13 (33%) CRT-D patients. The composite endpoint of death or time to first appropriate shock occurred in 25 (63%) CRT-D patients compared with 21 (37%) CRT-P patients (*p* = 0.013). Patients with CRT-P demonstrated a significantly greater improvement in left ventricular ejection fraction (LVEF) than those with CRT-D (15% vs. 9%, *p* < 0.003). Greater LVEF improvement was observed in patients with non-ischemic compared with ischemic cardiomyopathy (17% vs. 10%, *p* < 0.01). In multivariable analysis, pacemaker-induced cardiomyopathy (PICMP) was identified as a predictor of left ventricular (LV) reverse remodelling. *Conclusions*: In this prospective, non-randomized cohort, CRT upgrade was associated with long-term clinical and echocardiographic improvement. Differences observed between CRT-P and CRT-D groups should be interpreted cautiously, as the study was not designed for direct comparison. PICMP was independently associated with LV reverse remodelling.

## 1. Introduction

Nearly one-fourth of all cardiac resynchronization therapy (CRT) indications involve patients with previously implanted pacemakers or implantable cardioverter–defibrillators (ICDs) [1]. CRT upgrade is an established treatment for heart failure (HF) patients with reduced left ventricular ejection fraction (LVEF) and a high burden of right ventricular (RV) pacing who remain symptomatic despite optimal medical therapy [2].

Although RV pacing is beneficial in managing bradycardia, long-term RV pacing may disrupt normal ventricular activation, resemble left bundle branch block, and contribute to the development of HF [3,4,5,6]. Pacing-induced cardiomyopathy (PICMP), often leading to CRT upgrade, is potentially reversible through reverse remodelling after CRT implantation. Responders may subsequently no longer meet the criteria for ICD implantation for primary prevention [7,8,9,10]. Ischemic cardiomyopathy, characterized by structural myocardial damage, has been identified as a negative determinant of reverse remodelling in de novo CRT recipients [11,12,13] and is similarly associated with unfavourable outcomes in CRT upgrade populations [14,15]. While early randomized controlled trials generally excluded patients with pre-existing devices, the BUDAPEST-CRT Upgrade trial demonstrated that CRT upgrades improve survival, reduce HF hospitalization, and promote reverse remodelling over 12 months [16]. Nevertheless, data on long-term outcomes following CRT upgrades remain scarce.

This study aims to evaluate the long-term impact of CRT upgrades on clinical, laboratory, electrocardiographic, and echocardiographic parameters at a single tertiary care centre, and to identify predictors of reverse remodelling following upgrade.

## 2. Materials and Methods

### 2.1. Study Population

The study population included patients who underwent an upgrade to CRT between 1 October 2018 and 30 September 2020, at a single tertiary care centre, University Hospital Hradec Kralove. These patients were then prospectively followed. The study was conducted in accordance with the Declaration of Helsinki and was approved by the Ethics Committee of University Hospital Hradec Kralove. All participants provided written informed consent before the procedure and provided informed consent for data collection and analysis during a scheduled follow-up visit. Exclusion criteria included missed outpatient follow-up visits for any reason, refusal to provide informed consent, and incomplete data.

### 2.2. Data Collection

Baseline data collection prior to CRT upgrade comprised clinical assessment of functional capacity according to the New York Heart Association (NYHA) classification and a pre-implantation 12-lead electrocardiogram (ECG) for evaluation of underlying rhythm and QRS duration. Additional clinical data were extracted from the hospital information system, including comorbidities, the most recent coronary angiography results, and a complete transthoracic echocardiographic evaluation. Echocardiographic assessment included cardiac chamber dimensions, LVEF, regional wall motion abnormalities, valvular disease severity, and pulmonary hypertension grading. All examinations were performed according to a predefined core laboratory protocol and evaluated by a single experienced cardiologist to minimize variability. The same imaging modality and measurement approach were used at baseline and follow-up. LVEF was calculated using the Simpson biplane method when image quality was adequate; otherwise, it was visually estimated. The same methodology was consistently applied across all study time points. Laboratory assessments, including complete blood count and serum biochemistry (with NT-proBNP), were performed within seven days prior to CRT upgrade in a single central laboratory. Device interrogation data were collected either from the most recent outpatient follow-up or at bedside during hospitalization when CRT upgrade was indicated. CRT upgrade procedures were performed in a dedicated implantation laboratory by experienced electrophysiologists. Intra-procedural details, including device type (CRT-P or CRT-D), procedural duration, and left ventricular (LV) lead type and position, were recorded. Patients in whom transvenous left ventricular (LV) lead implantation failed were referred for surgical lead placement. The decision between CRT-P and CRT-D was made by the implanting physician in accordance with the latest clinical guidelines on cardiac pacing. The first routine device check occurred three months post-implantation, following local institutional protocol. Thereafter, patients with ICDs were evaluated every six months, while those with pacemakers were followed annually. A structured follow-up visit was conducted at 12 ± 2 months after CRT upgrade. This included reassessment of NYHA class, ECG, device interrogation, blood sampling, and transthoracic echocardiography, all performed by a single operator. Mortality data were obtained from regularly updated health insurance records.

### 2.3. Outcomes and Definitions

This study primarily aimed to assess the long-term clinical outcomes of patients undergoing CRT upgrade. The primary composite endpoint was defined as the time to all-cause mortality or first appropriate defibrillator therapy during follow-up. Because appropriate defibrillator therapy can occur only in patients with CRT-D, this composite endpoint is inherently asymmetric between device groups. Secondary objectives included evaluating the effects of CRT on electrocardiographic findings, humoral biomarkers, and echocardiographic parameters. Comparisons were made between CRT-P and CRT-D recipients, as well as between patients with ischemic cardiomyopathy (ICMP) and those with non-ischemic cardiomyopathy (NICMP). The study also sought to identify predictors of reverse remodelling. ICMP was defined as a history of myocardial infarction or the presence of myocardial scarring on transthoracic echocardiography associated with findings on invasive coronary angiography. PICMP was defined, following established criteria [10], as an LVEF < 50% with a ≥10% decrease from baseline in the presence of a right ventricular pacing burden of at least 40%. Alternative causes of LV dysfunction (ischemia, significant valvular disease, and other cardiomyopathies) were clinically excluded. RV pacing burden was assessed longitudinally during routine follow-up visits or hospitalizations for heart failure. The last device interrogation before CRT upgrade confirmed sustained high RV pacing exposure. Reverse remodelling was defined as an LVEF improvement of ≥10% after CRT upgrade.

### 2.4. Statistical Analysis

Continuous variables are expressed as median and interquartile range (IQR), because some parameters did not meet the assumption of normality. Categorical data are presented as numbers of patients and percentages. Normality was tested using the Shapiro–Wilk test. A Wilcoxon non-parametric paired test was held to compare medians of quantitative variables. McNemar’s symmetry test was used to assess qualitative parameters; the symmetry hypothesis was tested in the contingency table. To evaluate the difference in quantitative variables between groups, due to the non-fulfillment of the assumption of normality, non-parametric tests, Mann–Whitney or Kolmogorov–Smirnov, were used. Chi-square or Fisher’s exact test was used to determine the difference in qualitative variables. Lead position and reverse remodelling were compared using χ^2^/Fisher’s exact test with OR and 95% CI. To determine independent factors for LVEF improvement by ≥10%, a logistic regression model was performed. Clinical variables satisfying a threshold of *p* < 0.1 in univariate analysis were then re-entered into multivariate analysis. The level of significance α < 0.05 was considered statistically significant. All analyses were done using statistical software NCSS 2021.

## 3. Results

### 3.1. Patient Characteristics

A total of 97 patients (57 with CRT-P and 40 with CRT-D) were included in the study. Baseline characteristics are summarized in Table 1. The cohort was predominantly older, with a median age of 75 years (IQR 70–79), and predominantly male (*n* = 70, 72%). ICMP was present in 50 patients (52%). Arterial hypertension, atrial fibrillation, and chronic kidney disease (stage ≥ 3) were observed in more than half of the cohort. The median time from initial device implantation to CRT upgrade was 74 months (IQR 29–105), with a median right ventricular (RV) pacing burden of 97% (IQR 73–100%) on pre-procedural device interrogation. Of the 97 patients, 73 had a previously implanted pacemaker, and 24 had an ICD. All patients with ICDs were upgraded to CRT-D. Of the 73 pacemaker patients, 57 received an upgrade to CRT-P, and the remaining 16 received an upgrade to CRT-D; see Figure S1. The most common indication for CRT upgrade was PICMP, observed in 60 patients (62%). Additionally, eight patients (8%) underwent CRT upgrade with atrioventricular junction ablation for uncontrolled atrial fibrillation with rapid ventricular response, and 29 patients (30%) were upgraded due to progressive heart failure symptoms. LV lead implantation strategy was determined at the operator’s discretion, with the preferred target being the lateral or posterolateral branch of the coronary sinus. In one patient (1%), the LV lead was implanted via a thoracoscopic approach. A quadripolar lead was used in 90 (94%) patients, whereas a bipolar lead was implanted in 6 (6%) patients. The lead position was evaluated using standard fluoroscopic projections (LAO 30° and RAO 30°). In the LAO 30° projection, the LV lead was located in a lateral position in 82 patients (85%) and in a posterior position in 14 patients (15%), with no anterior placements observed. In the RAO 30° projection, 87 patients (91%) had a mid-ventricular position, 3 (3%) had a basal position, and 6 (6%) had an anterior position. Reverse remodelling occurred in 37.8% of patients with lateral lead positioning compared with 66.7% in the non-lateral group (*p* = 0.04).

### 3.2. Long-Term Clinical Outcomes

**Figure 1:**
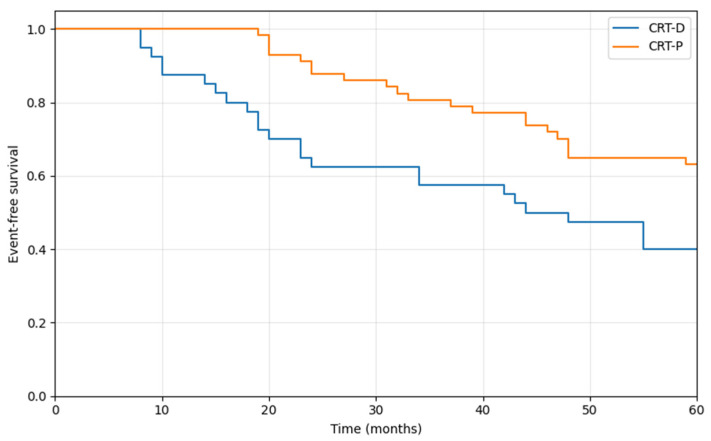
Kaplan–Meier graph of event-free survival (death or appropriate ICD therapy); *p* = 0.013. CRT-D = cardiac resynchronization therapy with defibrillator; CRT-P = cardiac resynchronization therapy with pacemaker.

Over the course of routine long-term clinical follow-up, 46 patients (47%) died from all causes. Appropriate device therapy was delivered in 13 patients (33%) within the CRT-D subgroup during the entire observation period. At five years follow-up, the composite endpoint of all-cause mortality or first appropriate defibrillator therapy occurred in 25 patients with CRT-D (63%) compared to 21 patients with CRT-P (37%), representing a statistically significant difference (*p* = 0.013; Figure 1). However, when stratified by etiology, there was no statistically significant difference in the occurrence of this composite outcome between patients with ischemic cardiomyopathy (54%) and those with non-ischemic cardiomyopathy (40%) (*p* = 0.181). However, the composite endpoint included appropriate ICD therapy, which could occur only in the CRT-D group. This creates a structural imbalance and may disadvantage the CRT-D group in comparisons of event-free survival.

### 3.3. Clinical, Laboratory, and Imaging Outcomes

**Figure 2:**
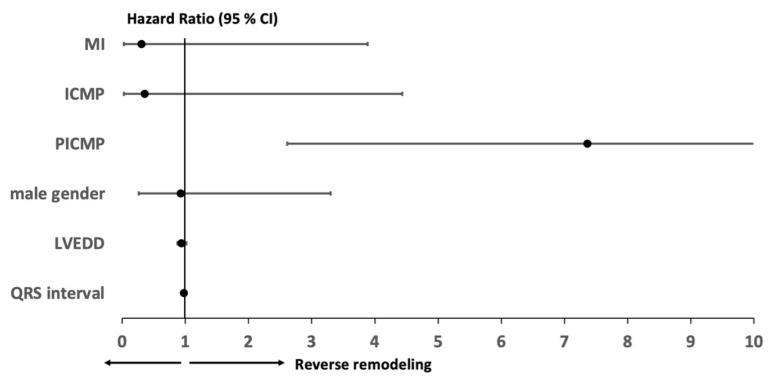
Forester plot of multivariate analysis for prediction of left ventricular improvement by at least 10%. MI = myocardial infarction, ICMP = ischemic cardiomyopathy, PICMP = pacing-induced cardiomyopathy, and LVEDD = left ventricular end-diastolic diameter.

The study outcomes are presented in Table 2. At the 12-month follow-up, 41% of patients demonstrated an improvement in NYHA functional class by at least one grade (*p* < 0.001). QRS duration was significantly reduced from a baseline median of 188 ms (IQR 174–200 ms) to 142 ms (IQR 132–154 ms) (*p* < 0.001). Similarly, NT-proBNP levels declined significantly, from 3920 ng/L (IQR 864–4890 ng/L) to 1177 ng/L (IQR 659–2436 ng/L) (*p* < 0.001). Transthoracic echocardiographic assessment revealed a significant increase in LVEF, from 25% (IQR 20–35%) at baseline to 43% (IQR 30–52%) at follow-up (*p* < 0.001). Additionally, left ventricular end-diastolic diameter (LVEDD) was significantly reduced from 59 mm (IQR 55–65 mm) to 57 mm (IQR 51–64 mm) (*p* = 0.004). A significant reduction in pulmonary hypertension was noted during the follow-up period (*p* < 0.001), while the severity of mitral regurgitation (MR) and tricuspid regurgitation (TR) remained unchanged (*p* = 0.193 and *p* = 0.646, respectively). Predictors of LVEF improvement by ≥10%, as determined by univariate and multivariate analyses, are detailed in Table S1. Multivariate analysis identified PICMP as the only independent predictor of reverse remodelling (Figure 2).

### 3.4. Subgroup Comparison

**Figure 3:**
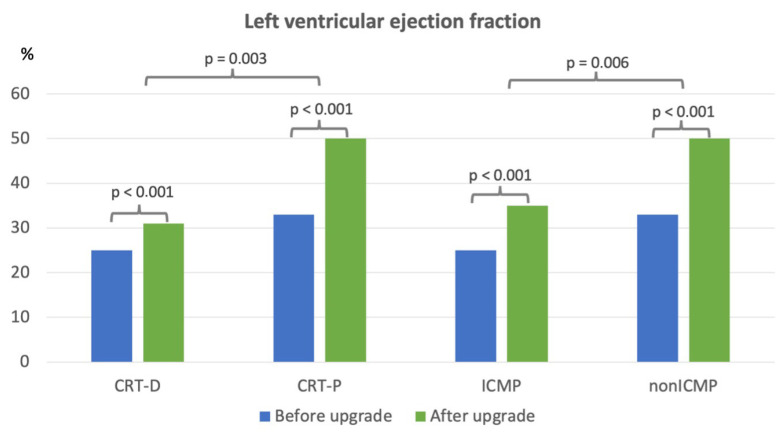
Graph of left ventricular ejection fraction results before and after the upgrade to cardiac resynchronization therapy. CRT-D = cardiac resynchronization therapy with defibrillator, CRT-P = cardiac resynchronization therapy with pacemaker, ICMP = ischemic cardiomyopathy, and non-ICMP = non-ischemic cardiomyopathy.

**Figure 4:**
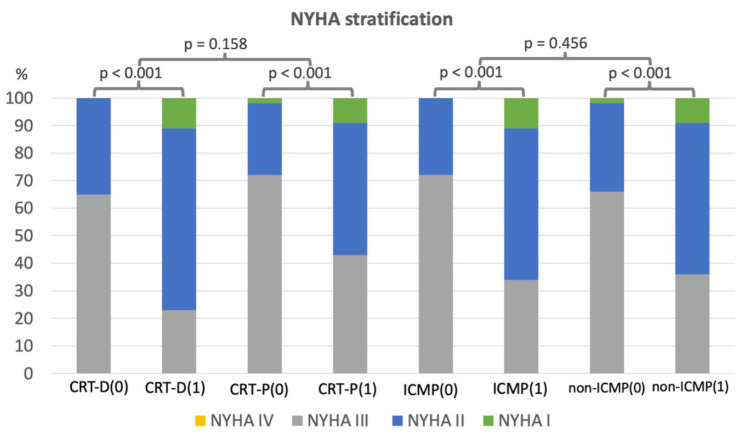
Graph of NYHA stratification before and after the upgrade to cardiac resynchronization therapy. CRT-D = cardiac resynchronization therapy with defibrillator, CRT-P = cardiac resynchronization therapy with pacemaker, ICMP = ischemic cardiomyopathy, non-ICMP = non-ischemic cardiomyopathy, (0) = before upgrade, and (1) = after upgrade.

As documented in Table 1, patients upgraded to CRT-D were more likely to be male, younger, and have a higher prevalence of ICMP. They exhibited lower rates of PICMP, atrial fibrillation, and use of anticoagulation compared with CRT-P recipients. At the one-year follow-up, both CRT-D and CRT-P groups showed significant improvements in NYHA functional class, with no statistically significant difference between groups (*p* = 0.158). A significant reduction in NT-proBNP was observed only in CRT-P patients, from a baseline median of 2129 ng/L (IQR 1016–4840 ng/L) to 1211 ng/L (IQR 864–2632 ng/L) (*p* = 0.012). Left ventricular ejection fraction (LVEF) improved significantly in both cohorts. CRT-P patients showed an increase from 33% (IQR 25–37%) to 50% (IQR 38–54%) (*p* < 0.001), while CRT-D patients improved from 25% (IQR 20–30%) to 31% (IQR 20–43%) (*p* < 0.001). The increase in LVEF was significantly greater in the CRT-P group (*p* = 0.003). In patients with ICMP, LVEF increased from 25% (IQR 20–33%) to 35% (IQR 25–47%) (*p* < 0.001), with no significant change in LVEDD (61 mm [IQR 57–67 mm] to 61 mm [IQR 56–67 mm]; *p* = 0.188). In contrast, patients with non-ICMP demonstrated significant improvements in both LVEF and LVEDD: LVEF increased from 33% (IQR 25–40%) to 50% (IQR 39–55%) (*p* < 0.001), and LVEDD decreased from 57 mm (IQR 53–60 mm) to 54 mm (IQR 49–59 mm) (*p* = 0.006). However, the magnitude of LVEF improvement was significantly greater in non-ICMP patients. Both ICMP and non-ICMP groups demonstrated similar improvements in NYHA class. Secondary outcome data are graphically presented in Figure 3 and Figure 4, with detailed statistical analyses provided in Tables S2 and S3.

### 3.5. Safety Outcomes

During the clinical follow-up period, three patients experienced significant device pocket hematomas, one of which necessitated surgical revision. Device extraction was required in six cases. Of these, four were due to infectious complications—either infective endocarditis or localized device pocket infection; one was related to a chronic ulcer at the pocket site, and one was attributed to a non-functional LV lead. Atrial lead replacement was performed in one patient due to lead failure. Additionally, five patients underwent LV lead replacement owing to loss of pacing capture during long-term follow-up.

## 4. Discussion

This prospective, single-centre, non-randomized study evaluated patients after CRT upgrade. At 12 months, patients showed significant improvement in functional status and electrocardiographic, biochemical, and echocardiographic parameters. Long-term follow-up focused on mortality, appropriate ICD therapy, and device-related complications. CRT-P patients compared with CRT-D showed a greater reverse remodelling, which could be explained by the heterogeneity of both groups in this real-world design. Notably, both ICMP and non-ICMP patients exhibited similar improvement in NYHA functional class; however, LVEF improvement was significantly greater in the non-ICMP group. PICMP emerged as the only independent predictor of reverse remodelling after CRT upgrade.

Upgrades from pacemakers or ICDs represent an incremental part of CRT indications; according to the last survey, the number of upgrades increased to 28% of all CRT procedures [1]. Despite being frequently compared with de novo CRT recipients, upgrade patients constitute a more heterogeneous group: they are typically older, predominantly male, and have a higher burden of comorbidities such as ischemic heart disease, valvular disorders, atrial fibrillation, chronic kidney disease, and anemia [1,17,18]. Moreover, these patients are less likely to receive guideline-directed medical therapy, including angiotensin-converting enzyme inhibitors (ACEis) and mineralocorticoid receptor antagonists (MRAs). Despite these differences, meta-analyses have shown similar benefits from CRT in both groups, including improved survival, LVEF, and NYHA class [19]. The BUDAPEST CRT Upgrade trial further supported CRT-D over ICD alone in reducing mortality, HF hospitalizations and LV reverse remodelling [16]. This effect was observed independently in patients with sinus rhythm and atrial fibrillation. Improvements in echocardiographic parameters following CRT upgrade have been confirmed in meta-analyses. One analysis reported a mean LVEF increase of 8% from a baseline of 26% [20]. In PICMP populations, the improvement was greater, ranging from 11% to 19% [10]. This difference likely reflects the reversibility of ventricular dysfunction in the absence of significant structural myocardial damage. In our cohort, median LVEF improved from 25% to 43%, likely driven by the high proportion of patients with potentially reversible cardiomyopathy: 62% had PICMP and 10% had arrhythmia-induced cardiomyopathy managed with CRT and AV junction ablation. However, CRT upgrade did not significantly affect MR or TR severity in this study. Although isolated reports describe MR reduction after biventricular pacing even in patients with preserved LVEF [21,22]. The mechanism of MR is complex and may be explained by adverse LV remodelling after RV pacing that results in functional MR [23]. The modest median reduction in LVEDD (2 mm), though statistically significant, may have been insufficient for clinical improvement in MR. Additionally, the high proportion of patients with prior valvular procedures may have limited the response. TR is less responsive to CRT, often being related to mechanical interference from the RV lead [23]. In our cohort, 41% of patients showed at least one-grade improvement in NYHA class, slightly lower than previous trials reporting 53% and 57% [24,25]. Despite the benefits of CRT, a substantial proportion of patients remain non-responders. QRS narrowing was found to be a predictor of reverse remodelling [26,27,28], and non-ICMP etiology has consistently been associated with better outcomes [11,12,13,14,15]. In our study, non-ICMP patients had significantly greater LVEF improvement than those with ICMP. Structural myocardial damage, such as scar and fibrosis, may limit the potential for LV reverse remodelling. Still, the absence of remodelling does not preclude symptomatic benefit, as NYHA improvement was observed similarly between groups CRT-D and CRT-P.

Identifying patients who benefit from CRT-D versus CRT-P remains clinically important. A key question is whether CRT-D offers added value in patients without prior ventricular arrhythmias. Bara et al. reported no survival benefit from ICD addition in PICMP patients without documented arrhythmias [29], whereas Leyva et al. found CRT-D to be superior in an unselected group of patients with structural heart disease, particularly ischemic cardiomyopathy, with lower cardiac mortality [30]. These findings suggest the benefit of CRT-D may depend on underlying etiology, especially as patients with ischemic cardiomyopathy may benefit more from the addition of an ICD. In the present study, long-term outcomes appear to have been predominantly driven by underlying myocardial substrate and indication-related selection rather than device type per se. Patients receiving CRT-D had a significantly higher prevalence of ischemic cardiomyopathy and structural heart disease, representing a population with intrinsically higher arrhythmic risk and more advanced myocardial damage. Conversely, the CRT-P group consisted largely of patients with pacing-induced cardiomyopathy, a potentially reversible condition with a more favourable remodelling profile. The higher incidence of the composite endpoint in the CRT-D group is therefore most plausibly explained by confounding by indication and baseline risk imbalance. Moreover, because the composite endpoint included appropriate ICD therapy—an event that cannot occur in CRT-P recipients—the endpoint itself is structurally asymmetric. This inherent asymmetry may artificially disadvantage the CRT-D group in time-to-event analyses and preclude interpretation of the observed difference as evidence of device inferiority. Consequently, our findings should be interpreted within the framework of patient selection and baseline substrate rather than as a comparative evaluation of CRT-D versus CRT-P efficacy.

Our observed 47% mortality rate over more than five years aligns with the high comorbidity burden in this population and is consistent with the 35% event rate reported by Trenson et al. in shorter follow-up [31]. Complication rates were also comparable: 5% of patients required LV lead replacement, aligning with REPLACE registry findings [32]. Notably, infection-related device extractions occurred in 4% of patients, underscoring that CRT upgrade is a known independent risk factor for pacing system infections [33]. This is consistent with data from the Danish registry, which reported higher complication rates in upgrade procedures [34]. Regarding laboratory findings, NT-proBNP levels decreased significantly, in line with the observed clinical and echocardiographic improvement. In contrast, creatinine showed a small but statistically significant increase; however, the absolute difference was minimal and not clinically relevant. This change likely reflects the natural progression of chronic kidney disease rather than an adverse effect of CRT upgrade. Hemoglobin levels remained stable during follow-up. Interestingly, non-lateral lead positioning was associated with a higher rate of reverse remodelling in our cohort. However, given the small size of the non-lateral subgroup, this finding should be interpreted with caution and may reflect sample variability rather than a true physiological effect. Therefore, this observation should be considered hypothesis-generating only, given the small subgroup size.

This study employed a prospective, non-randomized design; therefore, the findings should be interpreted with appropriate caution. The absence of a control group comprising patients who did not undergo CRT upgrade limits the ability to draw definitive conclusions regarding the efficacy of the intervention. Nevertheless, CRT upgrade indications were applied in accordance with contemporary guideline-based recommendations. In addition, conduction system pacing was not available at our institution during the study period, and as such, the study population consisted exclusively of patients receiving biventricular pacing. Future studies comparing CRT upgrade with conduction system pacing strategies, including His bundle or left bundle branch area pacing, may further refine patient selection and modify treatment algorithms. Due to the limited sample size, baseline differences between CRT-P and CRT-D groups, and the real-world design with individualized device selection, we did not perform propensity score matching or additional adjusted comparative analyses. Such an adjustment would have substantially reduced statistical power and might not have adequately addressed the inherent clinical selection process. Ischemic cardiomyopathy was defined based on transthoracic echocardiography and invasive coronary angiography. More precise characterization would ideally require cardiac magnetic resonance or nuclear imaging; however, these modalities were not performed due to limited availability and the presence of pre-existing CIEDs in our cohort.

## 5. Conclusions

The upgrade to cardiac resynchronization therapy was associated with significant improvements in functional capacity, biochemical and electrocardiographic parameters, and echocardiographic function. During the extended follow-up, differences were observed between CRT-P and CRT-D recipients in mortality and left ventricular reverse remodelling; however, these findings should be interpreted cautiously given the non-randomized design and baseline differences between groups. Pacing-induced cardiomyopathy (PICMP) was the only independent predictor of reverse left ventricular remodelling after CRT upgrade.

## Figures and Tables

**Table 1:** General characteristics of patients before the upgrade to cardiac resynchronization therapy. IQR = interquartile range, ICD = implantable cardioverter defibrillator, CRT-D = cardiac resynchronization therapy with defibrillator, CRT-P = cardiac resynchronization therapy with pacemaker, ACEi = ACE inhibitor, ARB = angiotensin receptor blocker, ARNI = angiotensin receptor and neprilisin inhibitor, MRA = mineralocorticoid receptor antagonist, DOACs = direct oral anticoagulants, and LMWH = low molecular weight heparin.

Characteristic	Entire Cohort (*n* = 97)	Cohort with CRT-D (*n* = 40)	Cohort with CRT-P (*n* = 57)	*p*
Age—years, median (IQR)	75 (70–79)	74 (68–76)	77 (72–80)	0.005
Female sex—*n* (%)	27 (28)	4 (10)	23 (40)	0.001
Body-mass index—median (IQR)	29 (26–33)	29 (26–32)	29 (26–33)	0.716
Ischemic cardiomyopathy—*n* (%)	50 (52)	33 (83)	17 (30)	<0.001
Arterial hypertension—*n* (%)	81 (84)	34 (85)	47 (82)	0.714
Atrial fibrillation—*n* (%)	67 (69)	22 (55)	45 (79)	0.012
paroxysmal—*n* (%)	19 (20)	8 (20)	11 (19)	0.563
persistent—*n* (%)	29 (30)	9 (23)	20 (35)	
permanent—*n* (%)	19 (20)	5 (13)	14 (25)	
Valvular heart disease—*n* (%)	30 (31)	11 (28)	19 (33)	0.541
Chronic obstructive pulmonary disease—*n* (%)	10 (10)	3 (8)	7 (12)	0.517
Diabetes mellitus—*n* (%)	44 (45)	22 (55)	22 (39)	0.110
Chronic kidney disease grade 3 and more—*n* (%)	57 (59)	21 (53)	36 (63)	0.305
Anemia—*n* (%)	39 (40)	16 (40)	23 (40)	0.972
Device characteristics				
Time to upgrade—months, median (IQR)	74 (29–105)	77 (33–106)	72 (27–104)	0.665
Ventricular pacing—%, median (IQR)	97 (73–100)	94 (27–99)	99 (90–100)	0.082
Pacemaker	73 (75)	16 (40)	57 (100)	<0.001
ICD	24 (25)	24 (60)	0	<0.001
Indication				
Pacing induced cardiomyopathy—*n* (%)	60 (62)	17 (43)	43 (75)	0.001
Progression of heart failure—*n* (%)	29 (30)	20 (50)	9 (16)	
Arrhythmia induced cardiomyopathy—*n* (%)	8 (8)	3 (8)	5 (9)	
Previous medication				
Betablocker—*n* (%)	84 (87)	38 (95)	46 (81)	0.067
ACEi/ARB/ARNI—*n* (%)	71 (73)	31 (78)	40 (70)	0.423
MRA—*n* (%)	46 (48)	23 (58)	23 (40)	0.096
Loop diuretic—*n* (%)	64 (67)	30 (75)	34 (60)	0.066
Amiodarone—*n* (%)	40 (41)	20 (50)	20 (35)	0.142
Antiplatelet drug—*n* (%)	45 (47)	27 (68)	18 (32)	<0.001
Anticoagulation—*n* (%)	65 (67)	21 (53)	44 (77)	0.016
DOACs—*n* (%)	23 (23)	4 (10)	19 (33)	0.091
Warfarin—*n* (%)	41 (43)	17 (43)	24 (42)	
LMWH—*n* (%)	1	0	1 (2)	

**Table 2:** Primary endpoints for all upgrade procedures. IQR = interquartile range, LVEF = left ventricular ejection fraction, LVEDD = left ventricular end diastolic diameter.

Parameter	Before Upgrade	1 Year After Upgrade	*p*-Value
Laboratory samplings			
Creatinin—mcg/L, median (IQR)	130 (94–156)	133 (96–166)	0.002
NTproBNP—ng/L, median (IQR)	3920 (864–4890)	1177 (659–2436)	0.004
Hemoglobin—g/L, median (IQR)	134 (124–147)	132 (122–142)	0.073
Electrocardiographic parameters			
QRS—ms, median (IQR)	188 (174–200)	142 (132–154)	<0.001
Echocardiographic parameters			
LVEF—%, median (IQR)	25 (20–35)	43 (30–52)	<0.001
LVEDD—mm, median (IQR)	59 (55–65)	57 (51–64)	0.004
	Device interrogation		
Biventricular pacing—%, median (IQR)	-	99 (98–100)	
Biventricular pacing >95%, *n* (%)	-	91 (94)	
Mitral regurgitation			0.193
None	2 (2)	5 (5)	
Mild	31 (32)	37 (38)	
Moderate	38 (39)	36 (37)	
Severe	26 (27)	19 (20)	
Tricuspid regurgitation			0.646
None	5 (5)	4 (4)	
Mild	38 (39)	43 (44)	
Moderate	38 (39)	31 (32)	
Severe	16 (16)	19 (20)	
Pulmonary hypertension			<0.001
None	35 (36)	64 (66)	
Mild	35 (36)	24 (25)	
Moderate	24 (25)	5 (5)	
Severe	3 (3)	3 (3)	
NYHA classification			<0.001
NYHA I	1 (1)	9 (10)	
NYHA II	29 (31)	52 (55)	
NYHA III	64 (68)	33 (35)	
NYHA IV	0	0	

## Data Availability

The data will be made available upon reasonable request.

## Supplementary Materials

The following supporting information can be downloaded at: https://www.mdpi.com/article/10.3390/medicina62030513/s1. Figure S1: Previous devices and upgrade to cardiac resynchronization therapy; Table S1: Univariate and multivariate risk analysis of reverse remodelling (defined as left ventricle ejection fraction improvement by at least 10%); Table S2: Secondary endpoints: comparison between upgrades to CRT-D versus CRT-P; Table S3: Secondary endpoints: comparison between upgrades in patients with ischemic cardiomyopathy versus non-ischemic cardiomyopathy.

## Abbreviations

The following abbreviations are used in this manuscript:

CRT	cardiac resynchronization therapy
CRT-P	cardiac resynchronization therapy with pacemaker
CRT-D	cardiac resynchronization therapy with defibrillator
LVEF	left ventricular ejection fraction
PICMP	Pacing-induced cardiomyopathy
ICD	implantable cardioverter–defibrillator
HF	heart failure
RV	right ventricular
ICMP	ischemic cardiomyopathy
NYHA	New York Heart Association
ECG	electrocardiogram
LV	left ventricular
NICMP	non-ischemic cardiomyopathy
IQR	interquartile range
LVEDD	left ventricular end-diastolic diameter
MR	mitral regurgitation
TR	tricuspid regurgitation
