# Supplementary material for: Long Term Outcomes and Predictors of Reverse Remodelling After Cardiac Resynchronization Therapy Upgrade
Source: Medicina (Kaunas). 2026 Mar 10;62(3):513. doi: 10.3390/medicina62030513 (PMC13028553; doi:10.3390/medicina62030513)
Supplement: Supplementary file 1 [file medicina-62-00513-s001.zip › medicina-4143686-supplementary.pdf]

Upgrade to cardiac resynchronization therapy: 1-year prospective clinical assessment in a single tertiary center (HK-CRT upgrade)

Authors

Jakub Šimka[1*], Eva Čermáková[2], Rudolf Praus[1], Jiří Dokoupil[1], Jakub Stříteckýý[1], Luděk Haman[1], Filip Varhaník[1], Radek Pudil[1], Petr Pařízek[1]

[1] 1st Department of Internal Medicine Cardiology and angiology, University Hospital Hradec Králové and Faculty of Medicine in Hradec Králové, Charles University, Czech Republic

[2] Department of Medical Biophysics, Faculty of Medicine in Hradec Králové, Charles University

Supplementary materials

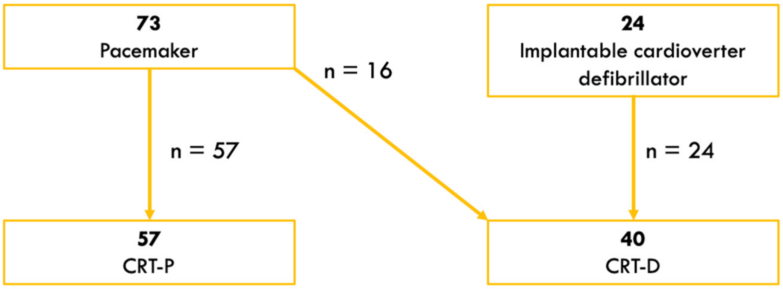

Figure S1. Previous devices and upgrade to cardiac resynchronization therapy.

CRT-D = cardiac resynchronization therapy with defibrillator, CRT-P = cardiac resynchronization therapy with pacemaker

	**Univariate analysis**		**Multivariate analysis**	
Covariate	Hazard ratio (95 % CI)	P	Hazard ratio (95 % CI)	P
Age at implantation	1.04 (0.99 – 1.10)	0.106		
Male gender	0.44 (0.17 – 1.18)	**0.091**	0.93 (0.26 – 3.30)	0.908
Time to upgrade	0.998 (0.99 – 1.01)	0.567		
Pacing induced cardiomyopathy	7.53 (2.99 – 18.95)	**<0.001**	7.36 (2.61 – 20.75)	**<0.001**
Ischemic cardiomyopathy	0.38 (0.16 – 0.89)	**0.023**	0.36 (0.03 – 4-43)	0.4
Myocardial infarction	2.01 (0.88 – 4.59)	**0.097**	0.31 (0.03 – 3.88)	0.336
Arterial hypertension	0.59 (0.20 – 1.73)	0.334		
Atrial fibrillation	0.78 (0.32 – 1.87)	0.576		
Valve disorder	1.57 (0.65 – 3.76)	0.314		
Chronic obstructive pulmonary disease	1.04 (0.27 – 3.96)	0.955		
Diabetes mellitus	1.62 (0.71 – 3.69)	0.248		
Anemia	0.79 (0.34 – 1.83)	0.587		
Chronic kidney disease	0.55 (0.24 – 1.27)	0.16		
QRS after upgrade	0.97 (0.94 – 1.00)	**0.025**	0.98 (0.95 – 1.01)	0.176
QRS difference	1.00 (0.98 – 1.01)	0.777		
Left ventricle ejection fraction	1.02 (0.98 – 1.07)	0.27		
Left ventricle end-diastolic diameter	0.92 (0.87 – 0.98)	**0.003**	0.942 (0.88 – 1.01)	0.109
ACEi or ARB	0.67 (0.27 – 1.67)	0.397		
MRA	1.41 (0.62 – 3.20)	0.397		
Betablocker	1.04 (0.31 – 3.44)	0.955		

Table S1. Univariate and multivariate risk analysis of reverse remodeling (defined as left ventricle ejection fraction improvement by at least 10 %)

ACEi = angiotensin converting enzyme inhibitor, ARB = angiotensin receptor blocker, MRA = mineralocorticoid receptor antagonist

Characteristic	CRT-D			CRT-P			p
	**Before upgrade**	**1-year after upgrade**	**p**	**Before upgrade**	**1-year after upgrade**	**p**	
**Laboratory samplings**							
Kreatinin - mcg/l, median (IQR)	133 (93 – 155)	141 (97 – 170)	**0.004**	127 (94 – 158)	124 ((96 – 166)	0.102	0.224
NTproBNP – ng/l, median (IQR)	1661 (788 – 4928)	1097 (578 – 2309)	0.192	2129 (1016 – 4840)	1211 (864 – 2632)	**0.012**	0.174
Hemoglobin – g/l, median (IQR)	141 (126 – 149)	137 (127 – 150)	0.788	134 (122 – 143)	131 (120 – 140)	**0.042**	0.294
**Electrocardiographic parameters**							
QRS – ms, median (IQR)	193 (175 – 203)	148 (138 – 162)	**<0.001**	180 (163 – 200)	138 (128 – 150)	**<0.001**	0.235
**Echocardiographic parameters**							
LVEF - %, median (IQR)	25 (20 – 30)	31 (20 – 43)	**<0.001**	33 (25 – 37)	50 (38 – 54)	**<0.001**	**0.003**
LVEDD – mm, median (IQR)	62 (57 – 68)	62 (55 – 68)	0.657	58 (54 – 61)	56 (50 – 60)	**<0.001**	**0.04**
**Mitral regurgitation - n (%)**							
None	2 (5)	4 (10)	0.473	0	1 (2)	0.107	0.437
Mild	14 (35)	15 (38)		17 (30)	22 (39)		
Moderate	17 (43)	12 (30)		21 (37)	24 (42)		
Severe	7 (18)	9 (23)		19 (33)	10 (17)		
**Tricuspid regurgitation - n (%)**							
• None	3 (8)	2 (5)	0.252	2 (4)	2 (4)	0.801	0.432
• Mild	19 (48)	24 (60)		19 (33)			
• Moderate	16 (40)	9 (23)		22 (39)			
• Severe	2 (5)	5 (13)		14 (25)			
**Pulmonary hypertension - n (%)**							
• None	17 (43)	30 (75)	**0.023**	18 (32)	34 (61)	**<0.001**	0.717
• Mild	14 (35)	5 (13)		21 (37)	19 (34)		
• Moderate	8 (20)	3 (8)		16 (28)	2 (4)		
• Severe	1 (3)	2 (5)		2 (4)	1 (2)		

NYHA classification							
• NYHA I – n (%)	0	4 (11)	**<0.001**	1 (2)	5 (9)	**<0.001**	0.158
• NYHA II – n (%)	14 (35)	25 (66)		15 (26)	27 (48)		
• NYHA III – n (%)	26 (65)	9 (24)		41 (72)	24 (43)		
• NYHA IV – n (%)	0	0		0	0		

Table S2. Secondary endpoints: comparison between upgrades to CRT-D versus CRT-P

IQR = interquartile range, LVEF = left ventricular ejection fraction, LVEDD = left ventricular end diastolic diameter, CRT-D = cardiac resynchronization therapy with defibrillator, CRT-P = cardiac resynchronization therapy with pacemaker

Characteristic	Ischemic cardiomyopathy			Non-ischemic cardiomyopathy			p
	**Before upgrade**	**1-year after upgrade**	**p**	**Before upgrade**	**1-year after upgrade**	**p**	
**Laboratory samplings**							
Kreatinin - mcg/l, median (IQR)	133 (95 – 161)	145 (110 – 172)	**<0.001**	127 (88 – 149)	114 (83 – 162)	0.804	**0.021**
NTproBNP – ng/l, median (IQR)	2039 (952 – 4915)	1201 (659 – 2414)	**0.026**	1991 (540 – 4564)	1144 (646 – 2477)	0.073	0.841
Hemoglobin – g/l, median (IQR)	134 (123 – 147)	137 (125 – 148)	0.750	136 (124 – 147)	131 (118 – 140)	**0.005**	**0.026**
**Electrocardiographic parameters**							
QRS – ms, median (IQR)	193 (172 – 205)	149 (136 – 163)	**<0.001**	180 (168 – 194)	140 (130 – 146)	**<0.001**	0.84
**Echocardiographic parameters**							
LVEF - %, median (IQR)	25 (20 – 33)	35 (25 – 47)	**<0.001**	33 (25 – 40)	50 (39 – 55)	**<0.001**	**0.006**
LVEDD – mm, median (IQR)	61 (57 – 67)	61 (56 – 67)	0.188	57 (53 – 60)	54 (49 – 59)	**0.006**	0.222
**Mitral regurgitation - n (%)**							
None	2 (4)	3 (6)	0.838	0	2 (4)	0.176	0.778
Mild	17 (34)	19 (38)		14 (30)	18 (38)		
Moderate	19 (38)	18 (36)		19 (40)	18 (38)		
Severe	12 (24)	10 (20)		14 (30)	9 (19)		
**Tricuspid regurgitation - n (%)**							
None	3 (6)	3 (6)	0.502	2 (4)	1 (2)	0.693	0.384
Mild	24 (48)	25 (50)		14 (30)	18 (38)		
Moderate	18 (36)	15 (30)		20 (43)	16 (34)		
Severe	5 (10)	7 (14)		11 (23)	12 (26)		
**Pulmonary hypertension - n (%)**							
None	20 (40)	35 (70)	**0.014**	15 (32)	29 (63)	**0.002**	0.182
Mild	14 (28)	10 (20)		21 (45)	14 (30)		
Moderate	13 (26)	4 (8)		11 (23)	1 (2)		
Severe	3 (6)	1 (2)		0	2 (4)		
**NYHA classification**							
NYHA I – n (%)	0	5 (11)	**<0.001**	1 (2)	4 (9)	**<0.001**	0.456

NYHA II – n (%)	14 (28)	26 (55)		15 (32)	26 (55)		
NYHA III – n (%)	36 (72)	16 (34)		31 (66)	17 (36)		
NYHA IV – n (%)	0	0		0	0		

Table S3. Secondary endpoints: comparison between upgrades in patients with ischemic cardiomyopathy versus non-ischemic cardiomyopathy

IQR = interquartile range, LVEF = left ventricular ejection fraction, LVEDD = left ventricular end diastolic diameter, CRT-D = cardiac resynchronization therapy with defibrillator, CRT-P = cardiac resynchronization therapy with pacemaker
